# Prediction of breast cancer-related lymphedema by dermal backflow detected with near-infrared fluorescence lymphatic imaging

**DOI:** 10.1007/s10549-022-06667-4

**Published:** 2022-07-10

**Authors:** Melissa B. Aldrich, John C. Rasmussen, Sarah M. DeSnyder, Wendy A. Woodward, Wenyaw Chan, Eva M. Sevick-Muraca, Elizabeth A. Mittendorf, Benjamin D. Smith, Michael C. Stauder, Eric A. Strom, George H. Perkins, Karen E. Hoffman, Melissa P. Mitchell, Carlos H. Barcenas, Lynn E. Isales, Simona F. Shaitelman

**Affiliations:** 1 Brown Foundation Institute of Molecular Medicine, McGovern Medical School, University of Texas Health Science Center at Houston, 1825 Pressler, 330D, Houston, TX 77030 USA; 2 University of Texas MD Anderson Cancer Center, 1515 Holcombe Boulevard, Unit 1502, Houston, TX 77030 USA; 3 Dana Farber/Brigham and Women’s Cancer Center, 450 Brookline Avenue, Boston, MA 02115 USA

**Keywords:** Breast cancer-related, Lymphedema, Near-infrared, Fluorescence, Lymphatic imaging, Lymphatics

## Abstract

**Purpose:**

Mild breast cancer-related lymphedema (BCRL) is clinically diagnosed as a 5%–10% increase in arm volume, typically measured no earlier than 3–6 months after locoregional treatment. Early BCRL treatment is associated with better outcomes, yet amid increasing evidence that lymphedema exists in a latent form, treatment is typically delayed until arm swelling is obvious. In this study, we investigated whether near-infrared fluorescence lymphatic imaging (NIRF-LI) surveillance could characterize early onset of peripheral lymphatic dysfunction as a predictor of BCRL.

**Methods:**

In a prospective, longitudinal cohort/observational study (NCT02949726), subjects with locally advanced breast cancer who received axillary lymph node dissection and regional nodal radiotherapy (RT) were followed serially, between 2016 and 2021, before surgery, 4–8 weeks after surgery, and 6, 12, and 18 months after RT. Arm volume was measured by perometry, and lymphatic (dys) function was assessed by NIRF-LI.

**Results:**

By 18 months after RT, 30 of 42 study subjects (71%) developed mild–moderate BCRL (i.e., ≥ 5% arm swelling relative to baseline), all manifested by “dermal backflow” of lymph into lymphatic capillaries or interstitial spaces. Dermal backflow had an 83% positive predictive value and 86% negative predictive value for BCRL, with a sensitivity of 97%, specificity of 50%, accuracy of 83%, positive likelihood ratio of 1.93, negative likelihood ratio of 0.07, and odds ratio of 29.00. Dermal backflow appeared on average 8.3 months, but up to 23 months, before the onset of mild BCRL.

**Conclusion:**

BCRL can be predicted by dermal backflow, which often appears months before arm swelling, enabling early treatment before the onset of edema and irreversible tissue changes.

**Supplementary Information:**

The online version contains supplementary material available at 10.1007/s10549-022-06667-4.

## Introduction

Breast cancer-related lymphedema (BCRL) is a chronic, incurable condition experienced by approximately 15%–40% of breast cancer survivors [1]. BCRL has been associated with patient-reported pain, depression, employment disability, and cellulitis with associated hospitalizations, all of which have significant socioeconomic costs and affect millions of breast cancer survivors worldwide [2–5]. Several factors contribute to the development of BCRL, including metastatic disease in regional lymph nodes (LNs) occluding normal lymphatic flow, as well as surgical and radiotherapy (RT)-induced interruption of lymphatic function in the treatment field [6–8]. These treatments cause the release of inflammatory cytokines that can arrest lymphangion pumping action [9, 10], a function that is essential for moving lymph and waste products from peripheral tissues [11, 12]. Without lymphangion activity to move lymph from the capillaries into collectors, regional lymph stasis appears as retention of fluid in interstitial spaces, and perhaps backward seepage from lymphatic collector vessels into lymphatic capillaries, and is commonly referred to as “dermal backflow.” Dermal backflow may be a compensation for lymphedema, or may be a harbinger of irreversible tissue changes, including accumulation of subcutaneous fat, immune cells, and the development of fibrosis [13].

No diagnostic method has been universally accepted for accurate detection of early changes in lymphatic function before the onset of irreversible BCRL tissue changes, yet early diagnosis may lead to the best chance of treatment efficacy [14–17]. Instead, the objective, albeit non-standardized, diagnostic criterion for cancer-acquired lymphedema is an increase in arm volume, which is typically measured no earlier than 3–6 months after locoregional treatment to avoid the transient swelling attributable to surgery and RT [18]. The most frequently used screening tools used for BCRL are self-reported symptoms, tape measure assessment of arm volume, and bioimpedance spectroscopy [19]. Bioimpedance measurement of increased tissue water content may provide a surrogate appraisal of lymphatic dysfunction in breast cancer survivors, but recent studies suggest that bioimpedance is sensitive only after the appearance of the clinical sign overt swelling, and even then, has a high false negative rate [20–23], although at least one study has reported positive outcomes when the device was used appropriately [24] There remains no accepted predictive sign or symptom indicating which patients will develop BCRL before it is grossly apparent by significant arm swelling. Moreover, risk reduction strategies including education, self-monitored use of compression sleeves, and exercise have failed to affect the incidence of BCRL [25]. A significant, unmet need thus exists to identify early-onset changes in the lymphatics, preceding irreversible lymphedema, for reliable use in a variety of practice settings to reduce the incidence of lymphedema and its sequelae that impair the quality of life of cancer survivors.

In this study, we longitudinally assessed arm lymphatics in patients with locally advanced breast cancer before surgery, after surgery, and up to 18 months after regional nodal RT, by using near-infrared fluorescence lymphatic imaging (NIRF-LI) to detect the first signs of lymphatic dysfunction. In prior studies, we showed that dermal backflow characterizes primary and secondary lymphedema and is not present in healthy individuals. Previous studies by us and others showed that NIRF-LI surveillance of breast cancer patients detected lymphatic dysfunction before overt arm swelling was noted [26–28]. In this study, we hypothesized that dermal backflow is an early harbinger of BCRL, can reliably predict the future onset of objectively diagnosed BCRL, and, in the future, could be used to direct the acute treatment of lymphatic dysfunction before chronic, irreversible tissue changes occur.

## Methods

### Study subjects and imaging

Signed informed consent was obtained from 60 study subjects (for 300 planned imaging study sessions) in a study conducted between 2016 and 2022 under the approval of the institutional review boards of both The University of Texas Health Science Center at Houston and The University of Texas MD Anderson Cancer Center (under FDA combinational investigational new drug application 106,345 for off-label intradermal administration of indocyanine green [ICG] dye with NIRF-LI [NCT 02949726]). Inclusion/exclusion criteria are listed in the study protocol (Supplementary material/appendix). Of the 60 study subjects consented, 18 were excluded from our analysis–seven passed away before completing the study (breast cancer), seven dropped out, one progressed to breast cancer on both sides, and three missed multiple visits due to SARS-CoV-2. Demographic and other data for the remaining 42 study subjects are shown in Table 1. Subjects with locally advanced breast cancer were longitudinally imaged with NIRF-LI to detect changes in lymphatic anatomy before axillary lymph node dissection (ALND), after ALND but before RT (given 4–8 weeks after surgery), and then at approximately 6 months, 12 months, and 18 months after RT. The imaging procedure consisted of two intradermal injections of 0.1 cm^3^/25 μg of ICG (Akorn, HUB Pharmaceuticals, or Diagnostic Green, LLC) in each dorsal hand and ventral/volar wrist for a total of eight aseptic injections (200 μg total ICG dose) at the start of each study session. Of note, no infections occurred due to ICG injection. Skin surfaces were illuminated with < 1·9 mW/cm^2^, 785 nm excitation light, and emitted fluorescence was captured by using a custom, variable focus and field of view (350–1900 cm^2^) NIRF-LI system described elsewhere [28]. ICG microdose injection and NIRF-LI was completed in 30–45 min to provide real-time images of lymphatic vessels or dermal backflow. Images were analyzed for presence of fluorescence outside of pulsing lymphatic vessels or lymph nodes (dermal backflow) using ImageJ software (ImageJ version 1.2.4, RRID: SCR_003070). For subject sessions that were interrupted due to SARS-2-Covid restrictions, yet perometer readings were available from other, routine checkup visits at the prescribed dates, the presence of dermal backflow was reported as present if detected at both the previous and immediately following study visits (9 of the 210 total study sessions).

**Table 1:** Patient demographics and baseline disease characteristics

Characteristic	Value
Age, year, median (range)	52 (28–68)
Race, *n* (%)	
Black	3 (7.1)
Other (Asian, American Indian/Alaska Native, multi-race)	6 (14.3)
White	33 (78.6)
Ethnicity, *n* (%)	
Hispanic or Latino	7 (16.7)
Non-Hispanic	35 (83.3)
Sex, *n* (%)	
Female	42 (100)
Male	0 (0)
Body mass index, mean (range)	29.3 (17.4–44.5)
Underweight (< 18.5), *n* (%)	1 (2.4)
Normal weight (18.5–24.9), *n* (%)	10 (23.8)
Overweight (25.0–29.9), *n* (%)	14 (33.3)
Obese (≥ 30.0), *n* (%)	17 (40.5)
Clinical T category, *n* (%)	
Tx	1 (2.3)
T1	3 (7.1)
T2	18 (42**·**9)
T3	10 (23.8)
T4b	4 (9.5)
T4d	6 (14.3)
Clinical N category, *n* (%)	
N1	18 (42.9)
N2	5 (11.9)
N3a	7 (16.7)
N3b	1 (2.4)
N3c	11 (26.2)
Neoadjuvant chemotherapy, *n* (%)	40 (95.2)
Taxanes, *n* (%)	39 (92.9)
Anthracyclines, *n* (%)	34 (81.0)
Number of lymph nodes removed at ALND, median (range)	24 (6–42)
Number of lymph nodes involved at ALND, median (range)	1 (1–36)
Lymphovascular space invasion, *n* (%)	9 (21.4)
Extracapsular extension, *n* (%)	12 (28.5)
Lumpectomy, *n* (%)	11 (26.2)
Mastectomy, *n* (%)	31 (73.8)
Cumulative radiation dose, Gy, median	50
Total number of fractions of radiation, median	25

### Perometry and objective/clinical diagnosis of lymphedema

At each study visit, arm volume was calculated by averaging three measurements per arm, acquired with a horizontal Perometer 400NT (Perosystem) as described previously [29]. The formula used to calculate relative volume change (RVC) of each arm from baseline (before ALND) measurements was RVC = (A_2_U_1_)/(U_2_A_1_) − 1, where A_1_ and A_2_ are arm volumes on the affected (ipsilateral) side at two different time points, and U_1_ and U_2_ are arm volumes on the opposite, unaffected (contralateral) side [30]. Objective diagnoses of mild to moderate BCRL were made based on an RVC of ≥ 5% measured at 6 months after RT or thereafter, as compared with the before-ALND baseline [15, 31].

As standard of care at MD Anderson Cancer Center, subjects were informed of strategies for reducing the risk of lymphedema, which included exercise, by their primary oncology team and in most cases, also by physiotherapists. Daily use of compression garments was prescribed after an objective diagnosis of moderate lymphedema (typically RVC ≥ 10%). Subjects could view the NIRF-LI process in real time during the study, and were informed of any abnormalities found. In a few cases, subjects independently sought physiotherapy treatments for BCRL before diagnosis. One study subject received lymphovenous bypass (LVB) at the time of ALND (subject #50), and 3 subjects received both LVB and vascularized lymph node transplant (VLNT) between 6 and 12 months after RT (subject #5) or between 12 and 18 months after RT (subject #s 6 and 12). Study data were collected and managed using REDCap electronic data capture tools hosted at MD Anderson Cancer Center [32, 33].

### Statistical analysis

Accuracy, sensitivity, and specificity of NIRF-LI for BCRL were calculated as previously described [34]. Accuracy was calculated as the proportion of true positive results in the population. Sensitivity was calculated as the number of true positives divided by the sum of the numbers of true positives and false negatives. Specificity was calculated as the number of true negatives divided by the sum of the numbers of true negatives and false positives. Positive likelihood ratio was calculated as sensitivity divided by (100-specificity). Negative likelihood ratio was calculated as (100-sensitivity) divided by specificity. Positive predictive value was calculated as the number of true positives divided by the sum of the numbers of true positives and false positives, times 100. Negative predictive value was calculated as the number of true negatives divided by the sum of the numbers of true negatives and false negatives, times 100.

## Results

### Appearance of dermal backflow during lymphedema development

**Fig. 1:**
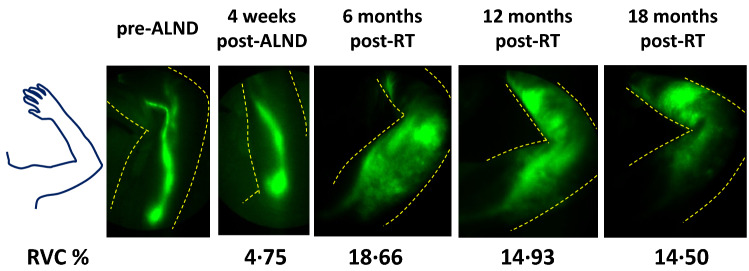
Near-infrared fluorescence lymphatic imaging (NIRF-LI) images of the left axilla (left to right) before axillary lymph node dissection (ALND), 4 weeks after ALND, 6 months after radiotherapy (RT), 12 months after RT, and 18 months after RT in study subject number 13. Dermal backflow appears as a cloudy dispersion of lymph (seen at 6, 12, and 18 months after RT), as opposed to linear lymphatic collector vessels (seen before and at 4 weeks after ALND). This study subject received physiotherapy for breast cancer-related lymphedema beginning at 6 months after RT. RVC, relative volume change (from baseline)

**Fig. 2:**
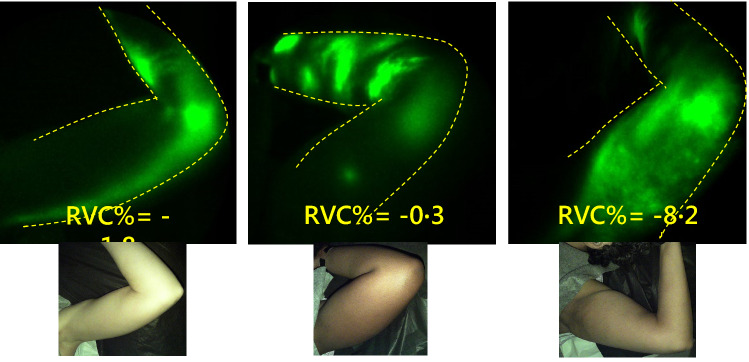
NIRF-LI images of the left axilla at 6 months after RT in study subject number 3 (left), number 12 (middle), and number 16 (right). Dermal backflow is apparent, despite the corresponding arm swelling/relative volume change (RVC%) values being − 1.8, − 0.3, and − 8.2 (left to right)

As an example of longitudinal NIRF-LI imaging, Fig. 1 illustrates the development and subsequent persistence of dermal backflow in study subject number 13. Her RVC values were 4.75% after surgery but before RT, 18.66% at 6 months after RT, 14.93% at 12 months after RT, and 14.50% at 18 months after RT, with extensive dermal backflow appearing at 6 months post-RT. Notably, dermal backflow often appeared months before the development of clinically diagnosable BCRL (RVC values ≥ 5%), as shown in Fig. 2, which provides NIRF-LI images for three separate study subjects (study subject numbers 3, 12, and 16), all displaying dermal backflow at 6 months after RT, even though all had RVC values below 5% at that time. All three subjects ultimately developed arm swelling of ≥ 5% at later study visits.

### *Longitudinal surveillance of backflow appearance and arm swelling of* ≥ *5%*

**Fig. 3:**
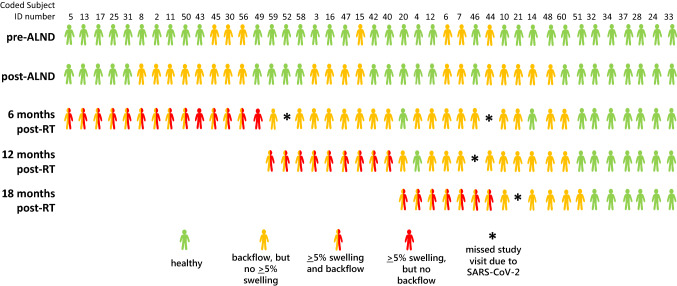
Modified swimmer plot denoting, at each surveillance visit, the absence of lymphatic dysfunction (green figures), appearance of dermal backflow (yellow), and objective diagnoses of breast cancer-related lymphedema (BCRL) from ≥ 5% RVC on the affected-side arm at 6 months after radiotherapy (RT) or later (red figures). Half-yellow/half-red figures represent subjects who displayed after-RT backflow and ≥ 5% swelling at the same visit. Asterisks denote clinical study interruption due to the SARS-CoV-2 pandemic. Blank spots after subjects developed BCRL/ ≥ 5% arm swelling do not represent subjects dropping out of the study—blank spots at 12 and 18 months post-RT allow quick visualization of BCRL onset at or after 6 months post-RT

A modified swimmer plot for all 42 study subjects denoting findings at each surveillance visit is shown in Fig. 3. A total of 36 study subjects (83%) presented with dermal backflow, and a total of 30 study subjects (71%) were objectively diagnosed with mild to moderate BCRL (i.e., ≥ 5% RVC) during this study. Dermal backflow was observed in all but one (subject #49) of the 30 BCRL subjects prior to or at the time of diagnosis. Of these 30 with BCRL, 14 were diagnosed 6 months after RT. In five of these 14 subjects, ≥ 5% RVC and dermal backflow were simultaneously first observed at 6 months post-RT. The long (8–10 month) window between post-surgical assessment and the first (6 months) post-RT surveillance session precluded any assessment of dermal backflow appearing before BCRL in these five subjects. Of these 14 subjects diagnosed 6 months after RT, eight had dermal backflow before or immediately after ALND (but before RT). Sixteen subjects were diagnosed with BCRL at 12 or 18 months after RT. All of these 16 subjects presented with backflow before BCRL. Conversely, six of the 36 subjects presenting with dermal backflow had not developed BCRL by the end of the study. Only six of 42 subjects did not present with dermal backflow or manifest BCRL during the course of this study. After excluding study subjects who had missed multiple study visits, including the 18-month follow-up, because of SARS-CoV-2, the mean delay between onset of dermal backflow and objective diagnosis of BCRL (by ≥ 5% RVC) was 8.3 months (range 0–23 months). Of note, blank spots on the plot at 12 and 18 months post-RT do not represent subjects that dropped out, but rather are left blank to allow quick visualization of BCRL onset at or after 6 months post-RT. Table 2 lists the diagnostic statistics for dermal backflow being an indicator, predictor, or both, of BCRL. Notably, backflow by NIRF-LI had an 83% positive predictive value and an 86% negative predictive value for the development of mild BCRL by 18 months after RT. The odds ratio for developing BCRL by 18 months after RT among subjects found to have dermal backflow by NIRF-LI was 29.00, with a positive likelihood ratio of 1.93, a negative likelihood ratio of 0·07, accuracy of 83%, sensitivity of 97%, and specificity of 50%. Notably, we observed unremarkable distribution of dermal backflow and BCRL by underweight, normal weight, overweight, and obese body mass index (BMI) status (Table 3).

**Table 2:** Statistics

Statistic	Value	95% CI
Sensitivity	96.67%	96.46 to 96.87%
Specificity	50.00%	49.10% to 50.91%
Positive likelihood ratio	1.93	1.89 to 1.97
Negative likelihood ratio	0.07	0.06 to 0.07
Positive predictive value	82.86%	82.46 to 83.26%
Negative predictive value	85.71%	84.89 to 86.54%
Accuracy	82.86%	71.46 to 94.26%
BCRL prevalence	71.43%	–
Backflow prevalence	83.33%	–

**Table 3:** Incidence of body mass index (BMI), breast cancer-related lymphedema (BCRL), and dermal backflow

BMI	Total	With backflow	With BCRL	With neither
Underweight	1	1 (100)	1 (100)	0 (0)
Normal weight	10	10 (100)	5 (50)	0 (0)
Overweight	14	11 (79)	11 (79)	3 (21)
Obese	17	13 (76)	12 (71)	3 (18)

Reported as Number (% of category)

## Discussion

In our study, dermal backflow was detected by NIRF-LI over eight months, on average, before the clinical development of BCRL and had a diagnostic odds ratio of 29 for the development of gross arm volumetric-assessed lymphedema. These results are consistent with lymphatic dysfunction as the initiating cause of lymphedema. The exact time frame between development of clinically measurable edema and irreversible changes that are not responsive to complete decongestive therapy (CDT) or reparative microsurgeries is unknown, but dermal backflow may be an effective and objective measure of lymphatic dysfunction at very early stages of BCRL progression. Significantly, these findings support the importance of early monitoring to implement physiotherapy to reverse dysfunction and restore fluid homeostasis, as suggested by non-imaging studies [14, 35] and shown by imaging in a NIRF-LI study of early head and neck cancer-related lymphedema treated with pneumatic compression therapy, in which dermal backflow disappeared or was reduced with physiotherapy [36].

Our study population was at particularly high risk of developing BCRL and, indeed, 19 subjects exhibited early-onset changes in the lymphatics, as visualized by NIRF-LI, before surveillance for lymphedema typically begins. Our cohort had aggressive local–regional disease with notable lymphatic metastatic burden, with 19 of 42 study subjects having cN3 disease. Notably, dermal backflow was seen in seven subjects at baseline (before surgery and RT), suggesting that factors other than ALND, such as neoadjuvant chemotherapy or cancer that has metastasized to lymph nodes, can influence BCRL development. Several reports have shown that receipt of doxorubicin and taxanes, both commonly prescribed as neoadjuvant chemotherapy for breast cancer, inhibits normal lymphatic flow [37, 38]. Also noteworthy is that in another eight of the 30 subjects diagnosed with BCRL, dermal backflow was first observed at the after-ALND-but-before-RT visit, which typically took place up to 10 months before surveillance for BCRL began (often scheduled for 3–6 months after the completion of RT), resulting in long lead times between observed lymphatic dysfunction and BCRL diagnosis with prescription of CDT. The early-onset changes visualized on NIRF-LI underscore the chronic duration of dysfunction that is associated with tissue changes that may become irreversible and render BCRL incurable.

Our study had some limitations. First, BCRL usually becomes evident within 18 months, but can manifest as late as decades, after cancer treatment, so some subjects may develop BCRL beyond our study timeline; thus, our false positive rate of 50% may reflect a substantial delay between the onset of dermal backflow and objective diagnosis. Second, our study cohort was too small to assess the effects of BMI on propensity to develop BCRL and BCRL temporal progression. Third, SARS-CoV-2 and its associated restrictions led to cancellation of some study visits, which confounded the accurate determination of backflow onset in some subjects. Fourth, the unique population studied (women with locally advanced breast cancer) was very well versed on risk reduction strategies and were offered therapeutic interventions as deemed clinically appropriate. Any use of prophylactic compression garments by those subjects who observed dermal backflow at imaging could have delayed or prevented the onset of BCRL-associated arm swelling and increased the number of false positives during the short timeframe of the program. As an example, at 6 months post-RT, study subject number 60 exhibited dermal backflow, but ≤ 5% RVC (no BCRL diagnosis). She started physiotherapy for BCRL prevention at that time, and at 12- and 18 months post-RT, although she still exhibited backflow, her RVCs were − 3.52 and 0.44%, respectively, at those visits (no BCRL diagnosis). Our study cohort included patients with particularly advanced nodal involvement, with 68% of patients having clinical N2–N3 disease at diagnosis and 14% with a diagnosis of inflammatory breast cancer. Our cohort’s cancer severity could explain why we observed only two subjects (#4 and #14) ever showing regression/reversal of backflow, compared to a previous study that observed 15/50 patients, with less advanced disease and no radiation treatment, reversing dermal backflow with compression sleeve use [26]. This general persistence of dermal backflow is consistent with our past longitudinal studies of head and neck cancer patients who also underwent lymph node dissection and radiation [39]. The four subjects in our study who underwent prophylactic or reparative microsurgeries did not display prevention or amelioration of BCRL. It is possible that the severity of our cohort’s breast cancers, or potential iatrogenic lymphatic damage from lymphatic microsurgeries, contributed to this result. NIRF-LI is considered an invasive procedure due to the intradermal injection of microdoses of ICG, and lymphedema patients are particularly concerned about possible infection risk. Aseptic injection technique was used in this study, and none of the subjects experienced infection due to ICG injections. Other BCRL surveillance methods, particularly limb measurement and BIS, are commonly used because they are inexpensive and noninvasive, but do not detect dermal backflow. The prospects for lymphedema “cure” will depend upon effective diagnosis at the first sign of lymphatic dysfunction to prevent progression from sub-clinical to symptomatic lymphedema. The impact of early detection on total healthcare costs for BCRL patients deserves more study [40, 41].

## Conclusions

Our findings suggest that BCRL can be predicted by using NIRF-LI surveillance for dermal backflow, which may precede the appearance of BCRL by over 8 months. Scheduling NIRF-LI surveillance at 3 months after RT could provide an earlier opportunity to predict BCRL prior to swelling. Starting complete decongestive therapy or restorative microsurgical treatment at the time of dermal backflow detection, before the development of mild BCRL and any irreversible tissue changes, may provide a unique opportunity to improve patient outcomes [42].

## Supplementary Information

Below is the link to the electronic supplementary material.

Supplementary file1 (DOCX 378 kb)

## Data Availability

Individual participant data that underlie the results reported in the article, after de-identification (text, tables, figures, and appendix), will be made available in case of reasonable request with investigators whose proposed use of the data has been approved by the authors.
